# Machine learning in medicine: a practical introduction to natural language processing

**DOI:** 10.1186/s12874-021-01347-1

**Published:** 2021-07-31

**Authors:** Conrad J. Harrison, Chris J. Sidey-Gibbons

**Affiliations:** 1 Nuffield Department of Orthopaedics, Rheumatology and Musculoskeletal Sciences, University of Oxford, Oxford, UK; 2 MD Anderson Center for INSPiRED Cancer Care, Department of Symptom Research, University of Texas MD Anderson Cancer Center, Houston, TX USA

## Abstract

**Background:**

Unstructured text, including medical records, patient feedback, and social media comments, can be a rich source of data for clinical research. Natural language processing (NLP) describes a set of techniques used to convert passages of written text into interpretable datasets that can be analysed by statistical and machine learning (ML) models. The purpose of this paper is to provide a practical introduction to contemporary techniques for the analysis of text-data, using freely-available software.

**Methods:**

We performed three NLP experiments using publicly-available data obtained from medicine review websites. First, we conducted lexicon-based sentiment analysis on open-text patient reviews of four drugs: Levothyroxine, Viagra, Oseltamivir and Apixaban. Next, we used unsupervised ML (latent Dirichlet allocation, LDA) to identify similar drugs in the dataset, based solely on their reviews. Finally, we developed three supervised ML algorithms to predict whether a drug review was associated with a positive or negative rating. These algorithms were: a regularised logistic regression, a support vector machine (SVM), and an artificial neural network (ANN). We compared the performance of these algorithms in terms of classification accuracy, area under the receiver operating characteristic curve (AUC), sensitivity and specificity.

**Results:**

Levothyroxine and Viagra were reviewed with a higher proportion of positive sentiments than Oseltamivir and Apixaban. One of the three LDA clusters clearly represented drugs used to treat mental health problems. A common theme suggested by this cluster was drugs taking weeks or months to work. Another cluster clearly represented drugs used as contraceptives. Supervised machine learning algorithms predicted positive or negative drug ratings with classification accuracies ranging from 0.664, 95% CI [0.608, 0.716] for the regularised regression to 0.720, 95% CI [0.664,0.776] for the SVM.

**Conclusions:**

In this paper, we present a conceptual overview of common techniques used to analyse large volumes of text, and provide reproducible code that can be readily applied to other research studies using open-source software.

**Supplementary Information:**

The online version contains supplementary material available at 10.1186/s12874-021-01347-1.

## Background

The last decade has seen an exponential increase in the volume of routinely collected data in healthcare [1]. As a result, techniques for handling and interpreting large datasets, including machine learning (ML), have become increasingly popular and are now very commonly referenced in the medical literature [2]. In some cases, these methods have demonstrated impressive performance in complex tasks such as image classification and the interpretation of natural language [3, 4]. But in many cases, ML algorithms do not demonstrate superior predictive performance to traditional statistical techniques [5–7], are poorly reported [8, 9], and raise concerns about interpretability and generalisability [10].

Clinicians are uniquely positioned to identify opportunities for ML to benefit patients, and healthcare systems will benefit from clinical academics who understand the potential, and the limitations, of contemporary data science [11]. Despite this, clinicians are seldom trained in big data analytics. The purpose of this article is to provide an introduction to the use of common machine learning techniques for analysing passages of written text.

Written text, for example medical records, patient feedback, assessments of doctors’ performance and social media comments, can be a rich source of data to aid clinical decision making and quality improvement. Where text-based data exist on the internet (for example, social media reviews of healthcare providers), it is technically possible to capture these using a process called web-scraping, which is straightforward to perform using open-source software [12]. Web-scraping software can be programmed to detect and download specific text from a website (e.g., comments on patient forums), and store these in databases, ready for analysis. This paper focuses on the analysis, rather than collection, of open text data, but readers wishing to scrape text from the internet should explore the rvest package [13], which is free to use. Before attempting web-scraping, it is important that researchers ensure they do not breach any privacy, copyright or intellectual property regulations, and have appropriate ethical approval to do so where necessary.

Often, these open-text datasets are so vast that it would be impractical to manually synthesise all of the useful information with qualitative research techniques. Natural language processing (NLP) describes a set of techniques used to convert passages of written text into interpretable datasets that can be analysed by statistical and machine learning models [4, 14].

### Lexicon-based sentiment analysis

Sentiment analysis is the process of assigning subjective meaning to words, phrases or other units of text [15]. Sentiment can be categorised simply as positive or negative, or can be related to more detailed themes, like the emotions that certain words reflect. Sentiment analysis serves a similar purpose to the process of ‘coding’ in qualitative research methods such as deductive thematic analysis [16]. A simple approach to sentiment analysis is to use a lexicon, which is a list of common words or phrases that have been matched to their categorical sentiment [17]. For example, a simple lexicon might match the words “love”, “favourite” and “respect” to a “positive” sentiment and the words “hate”, “pain”, and “anguish” to a “negative” sentiment. Lexicons serve as look-up tables that can automatically check the sentiment of each word or phrase in a passage of text. By quantifying the ratio of positive to negative sentiments in a sentence, for example, it is possible to start to understand the sentiment of the sentence overall. Lexicon-based sentiment analysis has been applied for detecting and monitoring disease outbreak based on the emotive sentiment of Twitter posts [18], assessing the public response to Obamacare [19], and to investigate patterns in social media posts about diabetes [20].

Sentiment analysis can be complicated by negation and sarcasm. For example, the word “not” reverses the sentiment of the word “recommend” in the sentence “I would not recommend this hospital to a friend or family member”. One potential way to handle this is by first splitting (tokenising) the sentence into bi-grams (pairs of adjacent words), rather than individual words [21]. This can help to identify words preceded by a negating particle and reverse their polarity, or sentiment can be assigned directly to the bi-gram [22]. In this case, the bi-gram “not recommend” might be assigned a negative sentiment. This approach to detecting negation has clear limitations in terms of sentence complexity, for example, negation in the sentence “the patient did not report a history of asthma” could not be handled by bi-grams. A more sophisticated and commonly used approach to handling negation is to employ algorithms that search for negation phrases. Examples include the NegEx algorithm [23] and its successor ConText [24], which can also qualify the *temporality* and *experiencer* of common medical conditions (i.e. whether a condition was present, when it was present, and in whom it was present). The sentiment of sarcastic remarks is often more dependent on context than the words themselves, and while attempts have been made to create sophisticated “sarcasm detectors”, this still poses a challenge to sentiment analysis [25].

Supervised and unsupervised ML algorithms can also be trained to assign sentiment to passages of text either independently, or with a lexicon as a hybrid approach. These approaches can account for complex interactions between words in a sentence more intricately than purely lexicon-based approaches. This paper demonstrates the simplest and least computationally intensive form sentiment analysis (the use of a publicly available lexicon only), but more advanced techniques have been described in detail elsewhere [26, 27].

### Unsupervised machine learning

Unsupervised ML algorithms aim to find previously undefined patterns within datasets, for example by grouping similar observations into clusters. They use data that have not been “labelled” by a human supervisor (i.e., observations which have not been categorised a priori) [14].

When applied to text analysis, unsupervised machine learning can be used to identify common themes within text by clustering words or sentiments that frequently appear together. This process is called “topic modelling” and is similar to an inductive thematic analysis [16, 28]. This approach has been applied to social media posts to understand common themes in a person’s reason for staying in, or leaving, an abusive relationship [29], discovering themes relating to drug non-compliance [30], and identifying health constructs that are important to patients, but not necessarily captured in patient-reported outcome measures [31].

### Supervised machine learning

Supervised machine learning algorithms aim to make predictions about an outcome (dependent variable) based on a set of features (independent variables). They use labelled datasets to learn how features and outcomes are related in order to predict outcomes from new, unlabelled, datasets [14]. They do this by iteratively adjusting their parameters to reduce prediction error in a training dataset, a process referred to as “tuning”. The performance of the algorithm is then usually tested with a second dataset, where it attempts to predict known outcomes from their associated features.

Broadly, supervised machine learning algorithms fall into two categories: regression algorithms and classification algorithms. Regression algorithms aim to predict a continuous outcome (e.g., blood pressure or risk of death) while classification algorithms (or classifiers) aim to predict categorical outcomes (e.g., positive or negative, benign or malignant). It is worth noting that in ML, the term “regression” is used slightly differently to traditional statistics, where regression can also be used to predict categorical outcomes. For example, a logistic regression model that aims to predict a binary outcome would be described as a classification algorithm rather than a regression algorithm, within ML literature [14].

Supervised ML algorithms have been combined with NLP to extract patient-centred outcomes from unstructured medical records [32], to detect emergent psychosis from language used by vulnerable youths [33], and to predict Care Quality Commission inspection results, based on hospitals’ social media comments [34].

### What this paper will achieve

This paper contains practical examples of common text analysis techniques, which we perform on a freely available dataset that contains over 200,000 patient drug reviews (in the form of text and an associated numerical rating, out of 10). The dataset is similar to what might be obtained by scraping online patient forums or healthcare provider review websites.

We provide code that can be modified and applied to similar analyses in other datasets. We aim to demonstrate to clinicians and qualitative researchers the type of text analyses that are easily performed with open source software, and provide practical understanding to academics that wish to apply these techniques in their own research.

### How to follow this paper

The methods section of this paper is structured into four parts, which in turn cover:

1. Basic NLP techniques for data cleaning in open-text datasets
2. Positive and negative sentiment analysis of drug reviews, with a freely-available lexicon
3. Unsupervised machine learning to identify similarities and differences between drugs, based on the words used to describe them
4. Supervised machine learning (classification) to predict whether a free text drug review will be associated with a dichotomised “Good” or “Bad” numerical score

We present samples of code written using the R Statistical Programming Language within the paper to illustrate the methods described, and provide the full script as a supplementary file. At points in the analysis, we deliberately simplify and shorten the dataset so that these analyses can be reproduced in reasonable time on a personal desktop or laptop, although this would clearly be suboptimal for original research studies.

While this paper is intended for readers who are relatively new to the field, some basic familiarity with the R programming language and machine learning concepts will make this manuscript easier to follow. Before attempting to recreate these experiments, readers may wish to read our introductory paper to machine learning in general, which covers R programming, supervised machine learning and model interpretation in more detail than is described in this paper [14].

## Methods

### Software, hardware and data

In recent years, the R and Python programming languages have become extremely popular for machine learning tasks [35]. They are both open-source, with thousands of free pre-programmed packages that can be used for statistical computing, and large online communities that provide support to novice users. R and Python have similar capabilities and are becoming increasingly interoperable, with many important machine learning packages now available for use in both languages.

We have performed these analyses using R version 4.0.3 in the RStudio computing environment. The experiments have been designed to run relatively quickly on a personal device. We used a 2017 MacBook Air, running macOS Mojave Version 10.14.6 on a 1.8 GHz processor with 8 GB of random-access memory.

In these experiments, we used the Drug Review Dataset from the University of California, Irvine Machine Learning Repository [36]. The dataset was obtained by scraping pharmaceutical review websites and contains drug names, free text patient reviews of the drugs, and a patient rating from 1 to 10 stars, among other variables. The dataset comes pre-split into training and test sets. We have randomly selected 5000 records from the training dataset to start with, in order to reduce computational demand.

### Data cleaning

In English text, many different combinations of characters can be used to mean the same thing. For example, “won’t”, “will not”, “Will not”, and “will not!” all use a different set of characters to convey very similar meanings. The main goal of data cleaning in NLP is to standardise text so that these variations are interpreted as the same feature by the machine learning models downstream.

There are a number of NLP techniques for standardising the free text comments [37]. We expanded contractions (e.g., replaced words “don’t” with “do not” and “won’t” with “will not”), removed non-alphanumeric characters, and converted all characters to lower case.

We also performed stemming with an English language stemmer. Stemming is the use of algorithms to reduce similar words to a common stem, for example by removing suffixes [38]. In our data cleaning pipeline, we have used the simple and freely available Porter algorithm for stemming, which largely works by removing inflexional suffixes. For example, the Porter algorithm would convert the words “learning”, “learned”, and “learns” to their common stem “learn” [39].

Next, we removed English “stop words” (common and usually unimportant words such as “the”, “and” and “is”) [40], and words with 3 or fewer characters. This dramatically reduces the number of features in the dataset, and allows algorithms to focus on the most meaningful elements of text. This stage of data cleaning is based on a principle known as Zipf’s Law, which states that the occurrence of a word within a body of text is inversely proportional to its rank in a frequency table. This means that the most commonly occurring word (often “the” in English language) occurs approximately twice as frequently as the second most common word, three times as frequently as the third most common word, and so on [41]. In keeping with Zipf’s law, 135 repeated words make up half of the one million words in the Brown University Standard Corpus of Present-Day American English [42]. For the linguistic analyses described in this paper, it is generally accepted that the most commonly used words are the least informative.

### Lexicon-based sentiment analysis

Next, we assigned a sentiment to each word in the dataset using a freely available lexicon known as “Bing”, first described for use in consumer marketing research by Minqing Hu and Bing Liu in 2004. The Bing lexicon ascribes either a “positive” or “negative” sentiment to 6786 different English words [17].

We chose to evaluate the overall sentiment of reviews for four different drugs, which we chose based on their diverse indications and pharmacological properties: Levothyroxine, Viagra, Oseltamivir and Apixaban. Levothyroxine and Viagra are used to treat hypothyroidism and erectile dysfunction respectively. Oseltamivir is used to treat and prevent influenza infections, and Apixaban is used to treat and prevent blood clots. Many people who take Levothyroxine and Viagra experience relief of troublesome symptoms, while people taking Oseltamivir or Apixaban prophylactically may not experience any appreciable benefit other than a lower risk of developing influenza or blood clots. We tested the hypothesis that Levothyroxine and Viagra are reviewed more positively than Oseltamivir and Apixaban.

To do this we tabulated the positive and negative sentiments assigned to all reviews of each drug, and calculated the percentage of sentiments that were positive.

### Unsupervised machine learning

Text data can be structured into a semantic hierarchy. A “token” is a series of characters that form the smallest semantic unit. In the experiments described in this paper, each word stem is its own token. If we had tokenised the drug reviews into bi-grams (to handle negation, for example), then each token would be two adjacent words. A “term” is a class of token with the same characters [38]. A “document” is a collection of tokens that appear together to convey a collective meaning, and a “corpus” is a collection of documents [37]. For example, within the corpus “Romeo and Juliet” the document “good night good night parting is such sweet sorrow that I shall say good night till it be morrow” might contain 19 tokens (words), with the term “night” appearing 3 times.

Before we can apply statistical or machine learning models to our text, we must first convert it into numeric data in a meaningful format. This can be achieved by creating a data table known as a document term matrix (DTM), sometime also referred to as a term document matrix (TDM) [14]. In the DTM, each row represents a document, and there is a column for each term used within the whole corpus. In a TDM, the orientation of rows and columns is switched. The cells contain numerals representing the number of times each term was used within a document. It is common for most cells in a DTM to contain the value “0”, as there are often many terms in a corpus, but these are not all used in each document.

**Fig. 1:**
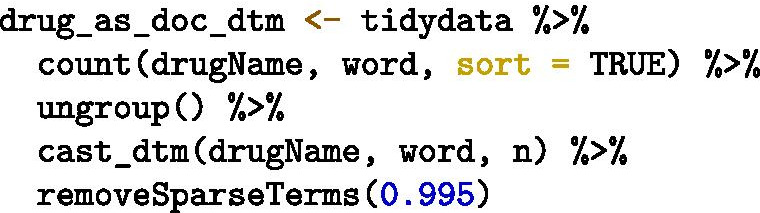
Creating a document term matrix from the data

**Fig. 2:**
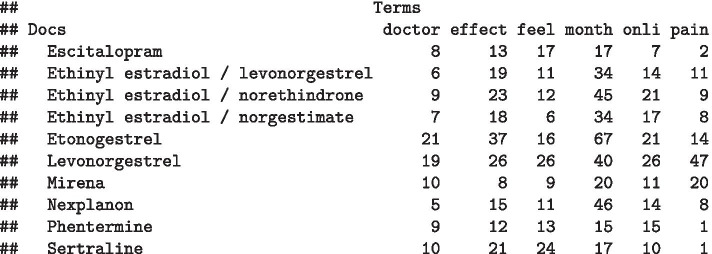
A part of the document term matrix

For our illustration of unsupervised machine learning, we chose to explore patterns between drugs based on the words used to describe them. To do this, we constructed a DTM where all the reviews of a given drug were combined into one document, representing the description of that drug by multiple reviewers. We then removed terms that were absent from ≥ 99.5% of documents (Fig. 1). The resulting DTM (Fig. 2) had 1111 different rows (i.e., 1111 different drugs, each representing a document) and 1948 columns (terms used within the corpus).

The values in our DTM represent term frequency, but it is also possible to weight these values by scaling them to account for the importance of a term within a document. A common way to do this, that readers should be familiar with, is the term frequency – inverse document frequency (TF-IDF) index. The term frequency is the number of times a term appears in a document. The inverse document frequency is the natural logarithm of the total number of documents, divided by the number of documents with a given term in it. The inverse document frequency gives an impression of the “importance” of a term within a corpus, by penalising common terms that are used in lots of documents. The TF-IDF is the product of the term frequency and the inverse document frequency, and provides insight into document-level semantics by measuring the frequency of a term within a document, and weighing this against the “importance” of the term within the corpus [38].

To cluster similar documents (drugs) together, we used an unsupervised machine learning technique called latent Dirichlet allocation (LDA). The LDA algorithm clusters terms into a predefined number of “topics” based on the probability of those terms being used together within a document. It then predicts which topic a document will belong to, based on the terms in that document.

Unlike other forms of clustering, such as k-means, it is possible for a term to belong to more than one topic in an LDA analysis [28]. This generally makes LDA a more appropriate tool for topic modelling, as in most cases it will be possible for a document to meaningfully belong to more than one topic. For example, the drug citalopram could belong to both a topic representing drugs that treat depression and to a topic representing drugs that cause nausea. We chose LDA as an easy-to-implement technique for basic topic modelling, although other methods have been described, including dynamic topic modelling (which maps topic structure within a corpus over time) [43], and structural topic modelling (which uses meta-data, such as author name and date to enhance cluster formation) [44].

**Fig. 3:**
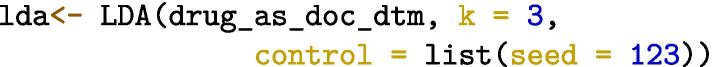
Latent Dirichlet allocation can be performed with a short passage of code

We programmed the LDA to cluster terms into 3 topics (Fig. 3). The number of topics was chosen for illustrative purposes. Choosing the number of clusters for an LDA-based topic model can be challenging. Where a number of clusters are expected based on an understanding of the corpus content, this number can be chosen (similarly to a deductive thematic analysis). Where the analysis is exploratory, the process can be repeated iteratively, and different models assessed for real-world plausibility. There are also statistical approaches to determining topic number, for example the rate of perplexity change, which relates to how well the model fits hold-out data [45].

To explore themes within the terms, we then identified the 10 terms most likely to belong to each topic. Next, we identified the 10 documents (drugs) most likely to belong to each topic. We hypothesised that similar drugs would be described in similar ways, and therefore cluster together.

### Supervised machine learning

Next, we rearranged the dataset into a DTM where each review was an individual document. Sparse terms were removed, resulting in 808 remaining features (terms), which were weighted by TF-IDF. We randomly selected a sampled 1000 reviews to further reduce computational burden. Clearly, researchers aiming to generate robust models should use as much data as possible, although this can add to the computing time and hardware requirements.

For each review, we took the associated star rating and dichotomised it into a binary outcome. A star rating of 1–5 was categorised as a “Bad” review and a star rating of 6–10 was categorised as a “Good” review. We then aimed to train 3 different supervised machine learning algorithms to predict whether a review was “Good” or “Bad”, depending on the words that were used in the review.

**Fig. 4:**
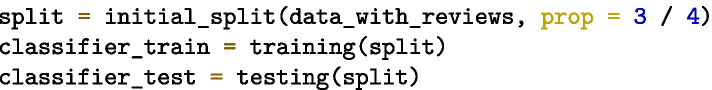
Splitting data into training and test sets

We split the data into training and test sets to create and evaluate our models respectively. We randomly assigned 75% of the reviews to the training set and 25% to the test set (Fig. 4).

This split resulted in a training dataset with 524 “Good” reviews and 226 “Bad” reviews. Training data with unbalanced classes can cause classifiers to predict the more frequently occurring class by default, particularly when sample sizes are small and features are numerous [46]. This can result in misleading accuracy statistics, for example if a model has a high sensitivity but poor specificity and is tested in a sample that has many more positive than negative observations.

To address the issue of class imbalance, we used the synthetic minority oversampling technique (SMOTE) [47]. The SMOTE algorithm creates new, simulated datapoints to balance the number of observations in each class. New data are simulated based on clusters that exist within the training data, using another form of machine learning algorithm known as K-nearest neighbours. Other methods to handle class imbalance are discussed elsewhere [48]. Briefly, these strategies involve oversampling the minority class, undersampling the majority class, or increasing the penalty for a majority class misspecification relative to a minority class misspecification.

The SMOTE algorithm transformed our training data into a dataset with 678 “Good” reviews and 678 “Bad” reviews. We then used this dataset to train 3 different types of supervised machine learning algorithm: a regularised logistic regression, a support vector machine (SVM), and an artificial neural network (ANN). These three types of classifier represent a spectrum of ML algorithms, ranging from relatively simple, easily interpretable and with a low number of parameters (regularised regression), to complex and difficult-to-interpret algorithms with a large number of parameters (ANN).

Regularised regression is similar to traditional regression, but applies an additional penalty term to each regression coefficient to minimise the impact of any individual feature on the overall model. Depending on the type of regularisation, and size of the penalty term, some coefficients can be shrunk to 0, effectively removing them from the model altogether. The purpose of regularisation is to prevent overfitting in datasets with many features [14].

Support vector machines aim to model a linear decision boundary (or “hyperplane”) that separates outcome classes in high-dimensional feature space. Model parameters can vary the way in which data are transformed into high-dimensional space, and how the decision boundary is drawn [14].

Artificial neural networks are so-called because they share a conceptual topography with the human central nervous system. They consist of interconnected neurons arranged in layers. Each neuron sums its inputs, multiplies this by a weight, and transforms the signal through an activation function. The weight of each neuron and their collective arrangement will affect model performance [14].

We would recommend that readers consult our previous instructional paper for a more thorough description of regularised regression, SVMs and ANNs [14]. For the purposes of this experiment, it is sufficient to understand that each model has a number of parameters which can be iteratively adjusted to improve that model’s predictive performance in samples of the training dataset.

In this study, model parameters were iteratively adjusted and tested across 10 bootstrap samples of the training dataset. When recreating this experiment, the number of bootstrap samples can be increased to improve model performance (reduce overfitting), but this will add to the computational demand. The parameters that maximised classification accuracy were chosen for the final models, which were then evaluated in the test dataset. Model performance was assessed with classification accuracy, area under the receiver operating characteristic curve (AUC) and confusion matrices.

## Results

### Lexicon-based sentiment analysis

The results of our sentiment analysis are displayed in Table 1. Levothyroxine and Viagra had a higher percentage of positive sentiments than Apixaban and Oseltamivir. The number of sentiments in the analysed dataset was low, and sentiments for each drug were negative overall.

**Table 1:** Sentiment analysis for reviews of Viagra, Levothyroxine, Oseltamivir and Apixaban

**Drug**	**Number of positive sentiments**	**Number of negative sentiments**	**Percentage of sentiments classified as positive**
**Viagra**	3	6	33%
**Levothyroxine**	7	16	30%
**Oseltamivir**	3	44	6%
**Apixaban**	0	2	0%

### Unsupervised machine learning

The ten terms most likely to appear in each topic are presented in Table 2, along with their beta values, which represent the term density within that topic. The five most common terms in Topic 1 (effect, feel, start, week, month) might suggest that this topic represents drugs that have taken weeks or months for the patient to feel its effects. Topic 1 also contains the terms “anxieti” and “depress” (note the Porter algorithm has reduced the term “anxieties” to the stem “anxieti”). This is interesting because many drugs used to treat anxiety and depression are associated with a gradual onset of action [49]. Topic 2 contains several terms that might relate to the female reproductive system. Topic 3 was less clearly defined.

**Table 2:** Terms most likely to belong to topics 1 and 2

**Topic 1**		**Topic 2**		**Topic 3**	
**Term**	**Beta**	**Term**	**Beta**	**Term**	**Beta**
effect	0.0180	period	0.0248	pain	0.0209
feel	0.0167	month	0.0238	effect	0.0162
start	0.0162	pill	0.0164	onli	0.0120
week	0.0135	control	0.0144	time	0.0117
month	0.0124	week	0.0142	start	0.0108
medic	0.0116	birth	0.0129	veri	0.0097
time	0.0115	weight	0.0122	week	0.0097
anxieti	0.0110	cramp	0.0121	doctor	0.0094
depress	0.0109	gain	0.0120	feel	0.0093
life	0.0101	start	0.0116	medic	0.0092

The ten documents (drugs) most likely to belong to each topic are presented in Table 3, along with their gamma values (the proportion of terms used in a document that belong to the given topic). In Topic 1, 9 of the 10 most common drugs are primarily used to treat mental health problems. The tenth, Levetiracetam, is an anti-epileptic. Interestingly, Levetiracetam’s use is associated with anxiety and suicidal ideation [50]. In Topic 2, all 10 drugs are hormone-based treatments primarily used as contraceptives. The drugs listed in Topic 3 did not have a clearly defined relation.

**Table 3:** Documents most likely to belong to topics 1 and 2

**Topic 1**		**Topic 2**		**Topic 3**	
**Document**	**Gamma**	**Document**	**Gamma**	**Document**	**Gamma**
Citalopram	0.9997	Etonogestrel	0.9999	Bisacodyl	0.9992
Prozac	0.9995	Nexplanon	0.9999	Clindamycin	0.9989
Pristiq	0.9994	Ethinyl estradiol / norgestimate	0.9998	Oseltamivir	0.9988
Vortioxetine	0.9993	Mirena	0.9998	Aluminium chloride hexahydrate	0.9988
Effexor	0.9992	Medroxyprogesterone	0.9997	Propofol	0.9982
Mirtazapine	0.9992	Skyla	0.9997	Polyethylene glycol 3350 with electrolytes	0.9981
Strattera	0.9990	Depo-Provera	0.9996	Bactrim DS	0.9980
Abilify	0.9990	Lo Loestrin Fe	0.9996	Otezla	0.9979
Aripiprazole	0.9989	Plan B	0.9995	MoviPrep	0.9979
Levetiracetam	0.9988	Desogestrel / ethinyl estradiol	0.9995	Levaquin	0.9977

### Supervised machine learning

The regularised regression and ANN took approximately 10 min each to train. The SVM took approximately 40 min to train. Model performance statistics are presented in Table 4. Classification accuracy ranged from 0.664, 95% CI [0.608, 0.716] for the regularised regression to 0.720, 95% CI [0.664, 0.776] for the SVM.

**Table 4:** Supervised machine learning algorithm performance

Model	Classification accuracy	AUC	Sensitivity	Specificity
Regularised regression	0.664, 95% CI [0.608, 0.716]	0.671, 95% CI [0.599, 0.734]	0.720, 95% CI [0.651, 0.785]	0.549, 95% CI [0.439, 0.651]
Support vector machine	0.720, 95% CI [0.664, 0.776]	0.725, 95% CI [0.658, 0.789]	0.815, 95% CI [0.755, 0.873]	0.524, 95% CI [0.420, 0.636]
Artificial neural network	0.688, 95% CI [0.628, 0.744]	0.672, 95% CI [0.599, 0.739]	0.982, 95% CI [0.959, 1.000]	0.085, 95% CI [0.026, 0.154]

*AUC* Area under the receiver operating characteristic curve, *CI* Confidence interval

## Discussion

In this paper, we have demonstrated techniques used to perform a range of common NLP tasks, and have provided annotated code which can be built upon and applied to other datasets (Supplementary Material). We were able to compare the sentiment of reviews for different drugs, accurately cluster similar medicines by the words used to describe them, and create models capable of determining whether a review was associated with a “Good” or “Bad” star rating, based on the language used. The dataset and techniques we have illustrated can be reimagined for a range of investigative purposes. For example, sentiment analysis could be used to analyse large volumes of free text data collected from patient experience surveys; topic modelling could be used to describe important health concerns discussed on patient forums or in qualitative interviews; and supervised ML algorithms could be applied to predict hospital performance from patients’ own words [34]. When applying these ML techniques to original research studies, we would recommend that authors adhere to appropriate methodological and reporting guidelines [51–54].

The primary purpose of this study was to provide a practical illustration of basic NLP techniques, and as such, there are notable limitations in the methods described. Firstly, in order to reduce the computational requirements of these tasks we sampled relatively small amounts of data from the Drug Review Dataset. In both statistics and ML, models with large numbers of independent variables (or features) require large sample sizes. Using small datasets, as we have done, increases the chance of model overfitting [55]. It would be important to externally validate our supervised ML algorithms in independent datasets.

We used relatively unsophisticated techniques for data cleaning. For example, we used stemming to remove inflexional suffixes. A limitation of this is illustrated in Table 2, where the term “anxieti” has been included in Topic 1. This stem does not capture the term “anxiety”. An alternative approach, lemmatisation, can reduce words to their base or dictionary form. This may be important, for example, where the base form of homonyms vary depending on whether the word is a verb or noun (e.g., the base form of the noun “saw” is “saw”, but the base form of the verb “saw” is “see”) [38]. Lemmatisation may yield better model performance than stemming [56]. We made no attempt to handle negation (e.g., by using the NegEx or ConText algorithms), or to explore more advanced NLP techniques such as named-entity recognition, relationship extraction, chunking or dependency parsing [4, 57].

Our supervised algorithms were relatively simple, and authors should consider incorporating other features into their training datasets. For example, we could have added columns to describe the sentiment of a review (based on the Bing lexicon), its lexical diversity, or its length in words or characters. When doing this, it is important to normalise the values of these features before algorithm training.

In this study, we used a decision threshold of 0.5 for our classifiers. In other words, the algorithms would classify a review as “Good” if they predicted the probability of it being “Good” as greater than 0.5. This threshold can be adapted for situations where either model sensitivity or specificity is particularly important.

There are many applications of web scraping, NLP and ML within healthcare and qualitative research. These techniques can be used to understand the health concerns of a population from social media, to process large volumes of medical records, or to qualify and quantify patient outcomes and experience from their own words. A basic understanding of these techniques will enable clinicians and qualitative researchers to work with data scientists, to identify areas of healthcare that could benefit from this technology.

## Conclusions

We have presented a practical introduction to common NLP techniques including data cleaning, sentiment analysis, thematic analysis with unsupervised ML, and predictive modelling with supervised ML. The code we have provided in the supplementary material can be readily applied to similarly structured datasets for a wide range of research applications.

## Supplementary Information

**Additional file 1.**

## Data Availability

The datasets generated and/or analysed during the current study are available in the University of California, Irvine Machine Learning Repository, https://archive.ics.uci.edu/ml/datasets/Drug+Review+Dataset+%28Drugs.com%29.

## Abbreviations

ANN: Artificial neural network
AUC: Area under the curve
DTM: Document term matrix
ML: Machine learning
NLP: Natural language processing
LDA: Latent Dirichlet allocation
SMOTE: Synthetic minority oversampling technique
SVM: Support vector machine
TDM: Term document matrix
TF-IDF: Term frequency inverse document frequency
