# Supplementary material for: Machine learning in medicine: a practical introduction to natural language processing
Source: BMC Med Res Methodol. 2021 Jul 31;21:158. doi: 10.1186/s12874-021-01347-1 (PMC8325804; doi:10.1186/s12874-021-01347-1)
Supplement: Supplementary file 1 — Additional file 1. [file 12874_2021_1347_MOESM1_ESM.pdf]

Machine learning in medicine: A practical introduction to natural language processing

Harrison CJ, Sidey-Gibbons CJ

01/05/2021

Download the dataset

Download the Drug Review Dataset from the University of California Irvine Machine Learning Repository by following this link.

Install and load packages

First, install and load the "devtools" package. This will allow you to choose the correct package versions.

```
install.packages("devtools")
library(devtools)
```

Make sure your working directory is set to the same folder that you have downloaded the "drugsCom_raw" folder to.

To reproduce the experiments described in this paper, you will need to install and load the following package versions. These may not be the latest versions, and RStudio may prompt you to install updates. If it does, proceed without updates by typing 3 into the console.

```
cran <- "http://cran.us.r-project.org"

install_version("tm", version = "0.7-8", repos = cran)
install_version("data.table", version = "1.14.0", repos = cran)
install_version("tidytext", version = "0.3.1", repos = cran)
install_version("dplyr", version = "1.0.6", repos = cran)
install_version("tidyr", version = "1.1.3", repos = cran)
install_version("topicmodels", version = "0.2-12", repos = cran)
install_version("performanceEstimation", verison = "1.1.0", repos = cran)
install_version("rsample", version = "0.1.0", repos = cran)
install_version("recipes", version = "0.1.16", repos = cran)
install_version("parsnip", version = "0.1.5", repos = cran)
install_version("workflows", version = "0.2.2", repos = cran)
install_version("tune", version = "0.1.4", repos = cran)
install_version("dials", version = "0.0.9", repos = cran)
install_version("keras", version = "2.4.0", repos = cran)
install_version("kernlab", version = "0.9-29", repos = cran)

library(tm)
library(data.table)
library(tidytext)
```

```r
library(dplyr)
library(tidyr)
library(topicmodels)
library(performanceEstimation)
library(rsample)
library(recipes)
library(parsnip)
library(workflows)
library(tune)
library(dials)
library(kernlab)
```

The drug Review Dataset is provided in two parts - training and test datasets. In order to make these experiments manageable for most personal devices, we will proceed with a random selection of 5000 drug reviews from the training dataset.

```r
training_data <- as.data.frame(fread("drugsCom_raw/drugsComTrain_raw.tsv"))
set.seed(123)
sample <- sample(nrow(training_data), 5000)
data <- training_data[sample,]
```

We can inspect the data using the dim( ) and head( ) functions. The dataframe should have 5000 rows and 7 columns.

```r
dim(data)
```

```
## [1] 5000    7
```

Data cleaning

Now, we create a function for that will perform the initial cleaning of the "review" column of the dataset.

```r
cleanText <- function(rawtext) {

  # As a result of the HTML-based web scraping, apostrophes appear in the text as
  # "&#039;""
  rawtext <- gsub("&#039;", "'", rawtext)

  # Expand contractions
  rawtext <- gsub("n't", " not", rawtext)
  rawtext <- gsub("won't", "will not", rawtext)
  rawtext <- gsub("wont", "will not", rawtext)
  rawtext <- gsub("'ll", " will", rawtext)
  rawtext <- gsub("can't", "can not", rawtext)
  rawtext <- gsub("cant", "can not", rawtext)
  rawtext <- gsub("didn't", "did not", rawtext)
  rawtext <- gsub("didnt", "did not", rawtext)
  rawtext <- gsub("'re", " are", rawtext)
  rawtext <- gsub("'ve", " have", rawtext)
  rawtext <- gsub("'d", " would", rawtext)
  rawtext <- gsub("'m", " am", rawtext)
  rawtext <- gsub("'s", "", rawtext)
```

```
  # Remove non-alphanumeric characters.
  rawtext <- gsub("[^a-zA-Z0-9 ]", " ", rawtext)

  # Convert all text to lower case.
  rawtext <- tolower(rawtext)

  # Stem words
  rawtext <- stemDocument(rawtext, language = "english")

  return(rawtext)
}


data$review <- sapply(data$review, cleanText)
```

We can now convert our data to the "tidy" format, where each row represents a unique word in a given reivew. In addition, here we remove stop words, and words with 3 or fewer characters.

```
tidydata <- data %>%
            unnest_tokens(word, review) %>%
            anti_join(stop_words) %>%
            distinct() %>%
            filter(nchar(word) > 3)
```

Sentiment analysis

Now we use the "Bing" lexicon to assign positive and negative sentiments to the words used to describe drugs in the dataset. We select four different drugs (Viagra, Oseltamivir, Apixaban and Levothyroxine), and count the positive and negative sentiments assigned to each review for that drug. We then calculate the percentage of sentiments that were positive for each drug.

```
drug_polarity <- tidydata %>%
  inner_join(get_sentiments("bing")) %>%
  filter(drugName == "Levothyroxine" |
         drugName == "Viagra" |
         drugName == "Apixaban" |
         drugName == "Oseltamivir") %>%
  count(sentiment, drugName) %>%
  pivot_wider(names_from = sentiment,
              values_from = n,
              values_fill = 0) %>%
  mutate(polarity = positive - negative,
         percent_positive = positive/(positive+negative) * 100) %>%
  arrange(desc(percent_positive))


drug_polarity
```

```
##        drugName negative positive polarity percent_positive
## 1        Viagra        6        3       -3        33.333333
## 2 Levothyroxine       16        7       -9        30.434783
## 3   Oseltamivir       44        3      -41         6.382979
## 4      Apixaban        2        0       -2         0.000000
```

Unsupervised machine learning (topic modelling)

For our illustration of unsupervised machine learning, we start by creating a document term matrix (DTM), where for each drug, all the reviews are combined into one document.

```
drug_as_doc_dtm <- tidydata %>%
  count(drugName, word, sort = TRUE) %>%
  ungroup() %>%
  cast_dtm(drugName, word, n) %>%
  removeSparseTerms(0.995)
```

We can visualise a sample of the DTM using the inspect function.

```
inspect(drug_as_doc_dtm)
```

```
##                                      Terms
## Docs                                  doctor effect feel month onli pain
##   Escitalopram                             8     13   17    17    7    2
##   Ethinyl estradiol / levonorgestrel       6     19   11    34   14   11
##   Ethinyl estradiol / norethindrone        9     23   12    45   21    9
##   Ethinyl estradiol / norgestimate         7     18    6    34   17    8
##   Etonogestrel                            21     37   16    67   21   14
##   Levonorgestrel                          19     26   26    40   26   47
##   Mirena                                  10      8    9    20   11   20
##   Nexplanon                                5     15   11    46   14    8
##   Phentermine                              9     12   13    15   15    1
##   Sertraline                              10     21   24    17   10    1
```

Next, we fit the LDA model to 3 clusters.

```
lda<- LDA(drug_as_doc_dtm, k = 3,
               control = list(seed = 123))
```

We can now examine the top terms used in each of the 3 topics, based on their beta value.

```
top_terms_per_topic <- lda %>%
                       tidy(matrix = "beta") %>%
                       group_by(topic) %>%
                       arrange(topic, desc(beta)) %>%
                       slice(seq_len(10))  # Number of words to display per topic

topic1 <- top_terms_per_topic[1:10,]
topic2 <- top_terms_per_topic[11:20,]
topic3 <- top_terms_per_topic[21:30,]

top_terms <- list(topic1, topic2, topic3)
top_terms
```

```
## [[1]]
##    topic    term       beta
## 1      1  effect 0.01795872
## 2      1    feel 0.01666886
## 3      1   start 0.01618559
## 4      1    week 0.01346453
```

```
## 5       1   month 0.01237980
## 6       1   medic 0.01160890
## 7       1    time 0.01151710
## 8       1 anxieti 0.01101236
## 9       1 depress 0.01086210
## 10      1    life 0.01011756
##
## [[2]]
##    topic    term       beta
## 1      2  period 0.02475260
## 2      2   month 0.02378735
## 3      2    pill 0.01640025
## 4      2 control 0.01443140
## 5      2    week 0.01424893
## 6      2   birth 0.01288171
## 7      2  weight 0.01216185
## 8      2   cramp 0.01209402
## 9      2    gain 0.01204094
## 10     2   start 0.01158890
##
## [[3]]
##    topic   term        beta
## 1      3   pain 0.020938682
## 2      3 effect 0.016166217
## 3      3   onli 0.011997526
## 4      3   time 0.011711224
## 5      3  start 0.010818266
## 6      3   veri 0.009739315
## 7      3   week 0.009739279
## 8      3 doctor 0.009400149
## 9      3   feel 0.009256581
## 10     3  medic 0.009208945
```

We can also look at the top documents per topic, based on their gamma value.

```
top_documents_per_topic <- lda %>%
                           tidy(matrix = "gamma") %>%
                           group_by(topic) %>%
                           arrange(topic, desc(gamma)) %>%
                           slice(seq_len(10))

topic1 <- top_documents_per_topic[1:10,]
topic2 <- top_documents_per_topic[11:20,]
topic3 <- top_documents_per_topic[21:30,]

top_docs <- list(topic1, topic2, topic3)
top_docs
```

```
## [[1]]
##         document topic     gamma
## 1     Citalopram     1 0.9996504
## 2         Prozac     1 0.9995328
## 3        Pristiq     1 0.9994135
## 4   Vortioxetine     1 0.9992665
```

```
## 5        Effexor     1 0.9992489
## 6    Mirtazapine     1 0.9992375
## 7      Strattera     1 0.9990401
## 8        Abilify     1 0.9989523
## 9   Aripiprazole     1 0.9988875
## 10 Levetiracetam     1 0.9988304
##
## [[2]]
##                            document topic     gamma
## 1                      Etonogestrel     2 0.9999001
## 2                         Nexplanon     2 0.9998605
## 3  Ethinyl estradiol / norgestimate     2 0.9997939
## 4                            Mirena     2 0.9997740
## 5               Medroxyprogesterone     2 0.9996899
## 6                             Skyla     2 0.9996821
## 7                      Depo-Provera     2 0.9996494
## 8                    Lo Loestrin Fe     2 0.9996214
## 9                            Plan B     2 0.9995399
## 10  Desogestrel / ethinyl estradiol     2 0.9995102
##
## [[3]]
##                                     document topic     gamma
## 1                                  Bisacodyl     3 0.9992445
## 2                                Clindamycin     3 0.9989695
## 3                                Oseltamivir     3 0.9988354
## 4              Aluminum chloride hexahydrate     3 0.9987911
## 5                                   Propofol     3 0.9982436
## 6  Polyethylene glycol 3350 with electrolytes     3 0.9981257
## 7                                 Bactrim DS     3 0.9979747
## 8                                     Otezla     3 0.9979414
## 9                                   MoviPrep     3 0.9979251
## 10                                  Levaquin     3 0.9977185
```

Supervised machine learning (predictive modelling)

We now create a dataframe to store the information that will be used to train and test our classification algorithms.

```
review_as_doc_dtm <- tidydata %>%
                     count(V1, word, sort = TRUE) %>%
                     ungroup() %>%
                     cast_dtm(V1, word, n) %>%
                     removeSparseTerms(0.995) %>%
                     weightTfIdf()
```

This creates a DTM where each review represents a single document, and sparse terms are removed. Term frequencies are weighted by inverse document frequency.

The following code randomly samples 1000 reviews (documents) in the DTM, and then matches them to their star ratings from the original data table. The star ratings are dichotomised as “Bad” or “Good”.

```
set.seed(123)

dataframe <- as.data.frame(as.matrix(review_as_doc_dtm))
```

```
classifier_sample <- sample(nrow(dataframe), 1000)
dataframe <- dataframe[classifier_sample,]

patient_number <- row.names(dataframe)
reviews <- rep(NA, nrow(dataframe))

for (i in 1:nrow(dataframe)){

  number <- patient_number[i]
  star_rating <- data[which(data$V1 == number),5]

  if (star_rating < 6){
    reviews[i] = "Bad"
      } else {
    if (star_rating > 5){
      reviews[i] = "Good"
    }
  }
}

data_with_reviews <- cbind(dataframe, reviews)
```

Now, we split the data into training and test sets.

```
split = initial_split(data_with_reviews, prop = 3 / 4)
classifier_train = training(split)
classifier_test = testing(split)
```

At this point, we check for class imbalance in our training data

```
table(classifier_train[,809])
```

```
##
##  Bad Good
##  226  524
```

From this table, we can see that there are more than twice as many "Good" reviews as "Bad" ones in the training data.

Here, we use the synthetic minority oversampling technique to address class imbalance in our training data. If you proceed without this step, you will find that the models tend to predict reviews in the test data as "Good", resulting in a high sensitivity but very low specificity.

```
balanced_training <- smote(reviews ~.,
                           classifier_train,
                           perc.over = 2,
                           perc.under = 1.5)
```

```
##
##  Bad Good
##  678  678
```

Next, we take bootstrap samples of the training data, and define our features and outcome.

```r
boots <- bootstraps(balanced_training, times = 10)
recipe <- recipe(reviews ~., data = balanced_training)
```

Regularised regression

The following code specifies which model parameters we want to tune in our bootstrap samples - in this case the penalty and mixture parameters.

```r
log_md <- logistic_reg(penalty = tune(),
                       mixture = tune()) %>%
          set_engine("glmnet")
```

Next, we create a table to store model performance statistics for each bootstrap sample, and specify a workflow for model tuning.

```r
penalty_mixture_grid <- grid_regular(penalty(),
                                     mixture(),
                                     levels = 10)

log_wfl <- workflow() %>%
           add_recipe(recipe) %>%
           add_model(log_md)
```

Now, we iteratively adjust our model parameters, and capture performance metrics in our table. Please note, this function may take about 10 minutes to run.

```r
penalty_mixture_fit <- tune_grid(log_wfl,
                                 resamples = boots,
                                 grid = penalty_mixture_grid)
```

We can examine the table to see which parameters performed best - here a penalty of 1.00e-10 and a mixture of 0.111 achieved the highest mean classification accuracy across our bootstraps (0.893).

```r
show_best(penalty_mixture_fit, metric = "accuracy") %>%
select(penalty, mixture, .metric, mean)
```

```
##        penalty   mixture  .metric     mean
## 1 1.000000e-10 0.1111111 accuracy 0.893065
## 2 1.291550e-09 0.1111111 accuracy 0.893065
## 3 1.668101e-08 0.1111111 accuracy 0.893065
## 4 2.154435e-07 0.1111111 accuracy 0.893065
## 5 2.782559e-06 0.1111111 accuracy 0.893065
```

Now, we choose the best parameters from our table, and add them to the workflow.

```r
best <- select_best(penalty_mixture_fit,
                   metric = "accuracy")

log_wfl <- finalize_workflow(log_wfl, best)
```

We can now apply the model to the test dataset, and collect performance statistics based on the predictions that it makes.

First, we write a function to extract our results. The following function will fit the workflow to the entire training dataset, then use it to collect predictions on the test dataset, before calculating 95% confidence intervals for our performance metrics through bootstrapping. The function also returns a confusion matrix.

```
performanceResults <- function(workflow, training_data, test_data){

  fitted_workflow <- workflow %>%
                     fit(training_data)
  # This fits the workflow to the training data.

  p <-  fitted_workflow %>%
        predict(new_data = test_data) %>%
        bind_cols(test_data %>% select(reviews))
  # This uses the fitted workflow to make predictions on the test data.

  # The next part specifies a function for extracting accuracy, sensitivity and
  # specificity.
  stats <- function(data, indicies) {

    preds <- data[indicies,]

    res <- c(
             preds %>%
             accuracy(as.factor(reviews), .pred_class) %>%
             select(.estimate) %>%
             as.numeric(),

             preds %>%
             sensitivity(as.factor(reviews), .pred_class, event_level = "second") %>%
             select(.estimate) %>%
             as.numeric(),

             preds %>%
             specificity(as.factor(reviews), .pred_class, event_level = "second") %>%
             select(.estimate) %>%
             as.numeric()
             )
  }

  # Now we calculate the statistics, based on 1000 bootstraps of the results.
  set.seed(123)
  bootstrap_est <- boot::boot(p, stats, R = 1000)

  # Here we generate confidence intervals, based on our bootstraps.
  acc <- bootstrap_est$t0[1]
  lower_acc <- boot::boot.ci(bootstrap_est, index = 1, type ="perc")$perc[[4]]
  upper_acc <- boot::boot.ci(bootstrap_est, index = 1, type ="perc")$perc[[5]]

  sens <- bootstrap_est$t0[2]
  lower_sens <- boot::boot.ci(bootstrap_est, index = 2, type ="perc")$perc[[4]]
  upper_sens <- boot::boot.ci(bootstrap_est, index = 2, type ="perc")$perc[[5]]

  spec <- bootstrap_est$t0[3]
```

```r
  lower_spec <- boot::boot.ci(bootstrap_est, index = 3, type ="perc")$perc[[4]]
  upper_spec <- boot::boot.ci(bootstrap_est, index = 3, type ="perc")$perc[[5]]

  # Next, we calculate the probability of each review belonging to the "Good" or "Bad"
  # class - this is used to calculate the area under the receiver operating
  # characteristic curve (AUC).
  auc_p <- fitted_workflow %>%
           predict(new_data = test_data, type = "prob") %>%
           bind_cols(classifier_test %>% select(reviews))

  # We specify a function for extracting the AUC.
  auc_boots <- function(auc_data, auc_indicies) {

    auc_preds <- auc_data[auc_indicies,]

    auc_preds %>%
      roc_auc(as.factor(reviews), .pred_Good, event_level = "second") %>%
      select(.estimate) %>%
      as.numeric()
  }

  # And here we repeat the bootstrap process for AUC.
  set.seed(123)
  bootstrap_auc_est <- boot::boot(auc_p, auc_boots, R = 1000)
  auc <- bootstrap_auc_est$t0[1]
  lower_auc <- boot::boot.ci(bootstrap_auc_est, index = 1, type ="perc")$perc[[4]]
  upper_auc <- boot::boot.ci(bootstrap_auc_est, index = 1, type ="perc")$perc[[5]]

  # Next, we combine our statistics and confidence intervals into a table, for reading
  # ease.
  colnames <- c("Statistic", "Value", "Lower 95% CI", "Upper 95% CI")
  row1 <- c("Accuracy", acc, lower_acc, upper_acc)
  row2 <- c("Sensitivity", sens, lower_sens, upper_sens)
  row3 <- c("Specificity", spec, lower_spec, upper_spec)
  row4 <- c("AUC", auc, lower_auc, upper_auc)

  results <- rbind(row1, row2, row3, row4)
  colnames(results) <- colnames
  results[,2:4] <- round(as.numeric(results[,2:4]), digits = 3)
  results <- as_tibble(results)

  # We also create a confusion matrix.
  p$reviews <- as.factor(p$reviews)

  conf_matrix <- p %>%
    conf_mat(truth = reviews, .pred_class)

  # Finally, we combine our results table and confusion matrix as a list.
  list <- list(results, conf_matrix)

  return(list)

}
```

This function only needs to be called once. From then on, it can be applied to each of our supervised machine learning workflows to extract all the results we need.

```
performanceResults(log_wfl, balanced_training, classifier_test)
```

```
## [[1]]
##     Statistic Value Lower 95% CI Upper 95% CI
## 1    Accuracy 0.664        0.608        0.716
## 2 Sensitivity  0.72        0.651        0.785
## 3 Specificity 0.549        0.439        0.651
## 4         AUC 0.671        0.599        0.734
##
## [[2]]
##           Truth
## Prediction Bad Good
##       Bad   45   47
##       Good  37  121
```

Support vector machine

We have already performed bootstrapping on our training data, and defined our outcome and features. The code to train and test the support vector machine and artificial neural network is very similar to that for the regularised regression. The only differences are the parameters that need tuning.

```
svm_md <- svm_poly(cost = tune(),
                   degree = tune()) %>%
           set_engine("kernlab") %>%
           set_mode("classification") %>%
           translate()

grid <- grid_regular(cost(),
                     degree(),
                     levels = 10)

svm_wfl <- workflow() %>%
           add_recipe(recipe) %>%
           add_model(svm_md)

svm_grid <- # This function may take 30-40 minutes to run.
          tune_grid(svm_wfl,
                    resamples = boots,
                    grid = grid)


best <- select_best(svm_grid,
                   metric = "accuracy")

svm_wfl <- finalize_workflow(svm_wfl, best)

performanceResults(svm_wfl, balanced_training, classifier_test)
```

```
## [[1]]
##     Statistic Value Lower 95% CI Upper 95% CI
## 1    Accuracy  0.72        0.664        0.776
```

```
## 2 Sensitivity 0.815        0.755        0.873
## 3 Specificity 0.524         0.42        0.636
## 4         AUC 0.725        0.658        0.789
##
## [[2]]
##           Truth
## Prediction Bad Good
##       Bad   43   31
##       Good  39  137
```

Artificial neural network

```
library(keras)
use_session_with_seed(123) # This code aims to make the neural network results more
# reproducible, however you may find that your model still achieves
# slightly different results due to randomness in certain aspects of
# model training.

nnet_md <- mlp(epochs = 10,
               hidden_units = tune(),
               dropout = 0.1) %>%
           set_engine("keras") %>%
           set_mode("classification") %>%
           translate()

grid <- grid_regular(hidden_units(),
                     levels = 10)

nnet_wfl <- workflow() %>%
            add_recipe(recipe) %>%
            add_model(nnet_md)

nnet_grid <- # This function may take 10 minutes to run.
             tune_grid(nnet_wfl,
                       resamples = boots,
                       grid = grid)

best <- select_best(nnet_grid,
                   metric = "accuracy")

nnet_wfl <- finalize_workflow(nnet_wfl, best)

performanceResults(nnet_wfl, balanced_training, classifier_test)
```

```
## [[1]]
##     Statistic Value Lower 95% CI Upper 95% CI
## 1    Accuracy 0.688        0.628        0.744
## 2 Sensitivity 0.982        0.959            1
## 3 Specificity 0.085        0.026        0.154
## 4         AUC 0.672        0.599        0.739
##
## [[2]]
##           Truth
## Prediction Bad Good
```

```
##        Bad    7    3
##        Good  75  165
```
